# *In Vivo* Osteogenic Potential of Biomimetic Hydroxyapatite/Collagen Microspheres: Comparison with Injectable Cement Pastes

**DOI:** 10.1371/journal.pone.0131188

**Published:** 2015-07-01

**Authors:** Erika Cuzmar, Roman A. Perez, Maria-Cristina Manzanares, Maria-Pau Ginebra, Jordi Franch

**Affiliations:** 1 Medicine and Animal Surgery Department, Autonomous University of Barcelona (UAB), V Building, 08193 Bellaterra, Spain; 2 Veterinarian Clinical Science Institute, Universidad Austral de Chile. Fundo Teja Norte, Valdivia, Chile; 3 Biomaterials, Biomechanics and Tissue Engineering Group, Department of Materials Science and Metallurgy, Technical University of Catalonia (UPC), Avda. Diagonal 647, E-08028 Barcelona, Spain; 4 Department of Nanobiomedical Science & BK21 PLUS NBM Global Research Center for Regenerative Medicine, Dankook University, Cheonan 330–714, Republic of Korea; 5 Institute of Tissue Regeneration Engineering (ITREN), Dankook University, Cheonan 330–714, Republic of Korea; 6 Human Anatomy and Embryology Unit, Pathology and Experimental Therapeutics Department, University of Barcelona (UB), C/ Feixa Llarga s/n, 08907, L’Hospitalet de Llobregat, Spain; Institute for Frontier Medical Sciences, Kyoto University, JAPAN

## Abstract

The osteogenic capacity of biomimetic calcium deficient hydroxyapatite microspheres with and without collagen obtained by emulsification of a calcium phosphate cement paste has been evaluated in an *in vivo* model, and compared with an injectable calcium phosphate cement with the same composition. The materials were implanted into a 5 mm defect in the femur condyle of rabbits, and bone formation was assessed after 1 and 3 months. The histological analysis revealed that the cements presented cellular activity only in the margins of the material, whereas each one of the individual microspheres was covered with osteogenic cells. Consequently, bone ingrowth was enhanced by the microspheres, with a tenfold increase compared to the cement, which was associated to the higher accessibility for the cells provided by the macroporous network between the microspheres, and the larger surface area available for osteoconduction. No significant differences were found in terms of bone formation associated with the presence of collagen in the materials, although a more extensive erosion of the collagen-containing microspheres was observed.

## Introduction

Increase of life expectancy as well as degenerative bone diseases make it essential for the development of promising biomaterials to restore or substitute damaged bone tissue. The gold standard for bone grafting is currently the fresh autologous cancellous bone graft since it possesses simultaneously osteoconductive, osteoinductive and osteogenic properties. Allografts and xenografts also provide osteoconductive and some potential osteoinductive properties. Nevertheless, the low availability and donor site morbidity in autografts and the possible transmission of disease and limited biological response in the allografts, limit their application [1–3]. For this reason, current efforts are centered in obtaining synthetic bone grafts that may enhance bone regeneration [4]. Among the different synthetic bone grafts, calcium phosphate biomaterials are the most widely available in the market. Moreover, injectable systems are being increasingly used in minimally invasive surgical procedures that reduce the time of surgery and the possible complications associated with them. Specifically, calcium phosphate cements combine their injectability with the ability to harden *in vivo*, producing after the setting reaction a nanostructured biomimetic hydroxyapatite, much more similar to the mineral phase of bone than the sintered hydroxyapatite products [5].

An additional advantage of this family of materials is their versatility, which allows combining them with numerous processing techniques [6]. We have recently shown that biomimetic calcium deficient hydroxyapatite microspheres can be obtained by emulsification of a calcium phosphate cement paste [7,8]. In this process, the stabilization of the microspheres is achieved through the setting reaction of the cement, which produces a calcium-deficient hydroxyapatite [9]. The microspheres obtained by emulsion of CPCs present several advantages compared to conventional ceramic calcium phosphate granules. First, they consist of a nanostructured calcium deficient hydroxyapatite, which is much closer to biological apatite than sintered calcium phosphate granules, with chemical and crystallographic similarities, and a high specific surface area [5,6]. In second place, the emulsion process allows combining the calcium phosphate phase with biopolymers and/or drugs, which can be incorporated not only on the surface, but also throughout the bulk of the microspheres [7,8]. This makes them good candidates not only for tissue regeneration and tissue engineering, but also as drug delivery vehicles with a homogenous release [10,11]. Specifically, we showed in a previous study that the addition of solubilized collagen in the cement paste not only was compatible with the emulsification process used to obtain microspheres, but it enhanced it, due to the anphiphilic properties of collagen [7].

The incorporation of collagen, either in the liquid or solid phase of calcium phosphate cements has been previously explored as a route to increase the biomimicry with bone extracellular matrix [12], and to enhance the handling properties [13], the mechanical properties [14–16] and the osteoblastic cell response [14,15]. Although the addition of biodegradable polymers in calcium phosphate cements has been claimed to be a good strategy to increase the *in vivo* resorption [12], the fact is that the effect of collagen on the *in vivo* performance of these biomimetic apatites obtained by a self-setting reaction remains to be investigated.

Therefore, the aim of this study was to assess the role of the spatial distribution of the material and the presence of collagen on the osteogenic capacity of a biomimetic calcium deficient hydroxyapatite. Thus, two specific objectives were targeted: i) to compare the bone regeneration potential of biomimetic hydroxyapatite microspheres obtained by emulsification of a calcium phosphate cement paste containing collagen or not, with that of an injectable apatitic calcium phosphate cement with the same composition; and ii) to assess the effect of collagen addition on the osteogenic potential of the biomimetic calcium deficient hydroxyapatite.

## Materials and Methods

### 2.1. Powder phase preparation

The powder phase of the cement was composed of α-tricalcium phosphate (α-TCP), obtained from the mixture of calcium hydrogen phosphate (CaHPO_4_, Sigma-Aldrich C7263)) and calcium carbonate (CaCO_3_, Sigma-Aldrich C4830) in a 2:1 molar ratio, sintered at 1400°C for 15 hours (Hobersal CNR-58) followed by quenching. The α-TCP was milled in a planetary ball mill (Pulverisette 6, Fritsch GmbB) to a median particle size of 2.44 ± 0.05 μm). 2 wt% of precipitated hydroxyapatite (PHA, Alco ref. 1.02143) was added as a seed. For the in vivo studies the CPC powder was sterilized with γ irradiation at 25 kGrays.

### 2.2. Production of calcium phosphate cements

Bovine type I collagen was obtained from bovine pericardium as described elsewhere [17]. Collagen pellets, sterilized by ethylene oxide, were dissolved in a 50 mM acetic acid aqueous solution at a concentration of 10 mg/ml. The acetic acid solution was previously sterilized by filtering through a 0.22 μm sterile filter (Millipore). The collagen solution and the powder were mixed at a liquid to powder ratio of 0.45 ml/g to obtain the collagen-containing cements (coded as coll-CPC) [15]. For the pristine calcium phosphate cements (coded as CPC), a 50 mM acetic acid solution was used as the liquid phase and mixed with the powder at the same liquid to powder ratio. For the *in vivo* study, the samples were prepared *in situ* prior to implantation.

### 2.3. Production of biomimetic hydroxyapatite microspheres

The MS were prepared by an emulsion technique. Ceramic suspensions were obtained by mixing the ceramic powder at a liquid to powder ratio of 0.8 mL/g, either with a 10 mg/ml collagen solution or with a 10x PBS solution, to obtain the collagen-containing MS (coded as MS-coll) or the pristine microspheres (coded as MS) respectively [7]. Three milliliters of the ceramic slurry were introduced into a beaker, which contained 300 mL of olive oil. An emulsion was formed by mixing both components with a mechanical stirrer (Heidolph BDC 2002), at 900 rpm, for a period of time equal to the setting time of the CPC paste. Afterwards, the microspheres were extracted by adding a 0.9% sodium chloride and 0.01% surfactant (Triton X-100) aqueous solution, and immersed in water for 7 days, in order to allow for the complete transformation of α-TCP into a calcium deficient hydroxyapatite. The microspheres were sieved between 100 and 400 μm. For the *in vivo* studies, the samples were sterilized with γ irradiation at 8 kGrays.

### 2.4. Material characterization

X-ray diffraction (XRD) was used to analyze the phases present in the cements and the microspheres after 7 days setting in water at 37°C. The cements and the microspheres were crushed and a powder diffraction pattern was obtained (Philips MRD, Cu K_α_,40 μA, 45 kV, scan step size 0.017°, step time 50 s). The peaks were indexed using cards JCPDS-29-359 for α-TCP and JCPDS-9-432 for apatite (Joint Committee on Powder Diffraction Standards, 1988). Scanning electron microscopy (SEM, JEOL JSM-840) was used to characterize the morphology and microstructure of the materials. Samples were coated with a thin layer of gold to increase the conductivity. Mercury intrusion porosimetry (MIP, Autopore IV Micromeritics) was performed to examine the porosity content and to determine the pore size distribution within the different samples.

### 2.5. *In vivo* study

The study was previously approved by the Ethics Comittee for Human and Animal Experimentation (CEEHA- Ref 2016) at the Autonomous University of Barcelona.

Bilateral femoral implantations were performed in thirty adult female New Zealand rabbits with a weight range of 4.8–5.5 kg and an age range of 8–12 months. Four different materials were implanted in the defects: calcium phosphate cement (CPC), calcium phosphate cement with collagen in the liquid phase (coll-CPC), calcium deficient hydroxyapatite microspheres (MS) and collagen-containing calcium deficient hydroxyapatite microspheres (coll-MS). As a control, a cavity defect with the same geometry was used where no material was implanted. The implantation times were 4 and 12 weeks, using 6 rabbits for each group. Two additional rabbits were used to assess the response of the four materials studied at an early implantation time of 12 days.

For surgical procedure, the animals were preanesthetized using buprenorphine (0.03mg/kg s.c.), midazolam (0.50 mg/kg s.c.) and medetomidine (0.05 mg/kg s.c). Anesthesia was induced with propofol (2,5 mg/kg i.v) and maintained with inhaled isoflorane (2%) in an oxygen carrier. The femoral regions of the rabbits were clipped and subsequently scrubbed with chlorhexidine gluconate solution 4% for an aseptic preparation of the surgical field. Both lateral aspects of the femoral condyles were exposed by a lateral parapatellar incision. A critical size defect of 5 mm diameter and 5mm depth was created in the condyle using drill bits of increasing size under copious irrigation with physiological saline. The CPC pastes were prepared *in situ* and implanted into the defect site. 100 mg of MS and coll-MS were combined with 1 mL of rabbit blood to make a blood clot, which was then implanted into the defect. After filling the defects, the joint capsule, the subcutaneous tissue and the skin were sutured in layers. The animals were euthanized at the scheduled survival times with an overdose of sodium pentobarbitone (200mg/kg/i.v.) according to the legislation of the American Veterinary Medical Association (AVMA). A pre-euthanasia sedation of midazolam (0.50 mg/kg s.c.) and medetomidine (0.05 mg/kg s.c) was used for animal welfare reasons.

### 2.6. Radiological analysis

Immediately after surgery, as well as after 4 and 12 weeks, femoral medio-lateral and cranio-caudal radiographs were taken to confirm the correct location of the implants.

### 2.7. Histological preparation of the samples

#### 2.7.1. Undecalcified samples

Tissue explants were fixed in 10% formaldehyde solution for one week. The samples were rinsed, trimmed of excess soft tissue and dehydrated in an increasing series of ethanol solutions (70–00%), and infiltrated with 4 different graded mixtures of ethanol and infiltrating resin (Technovit, 7200). The samples were then polymerized, sectioned and polished with 600, 800, 1200 and 2400 silicon carbide papers, prior to sputtering of the surface with carbon to allow for proper electrical conductivity for scanning electron microscopy analysis.

#### 2.7.2. Decalcified samples

Tissue explants from the 12 days implantation group were fixed in 10% formaldehyde solution for one week, and subsequently placed in a 5% nitric acid decalcifying solution for another week. Samples were then embedded in paraffin and semi-thin histological sections (15 μm) of the decalcified samples were obtained using a microtome (Jung Biocut 2035, Leica) and thereafter stained with hematoxilin and eosin.

### 2.8. Histology and histomorphometry

#### 2.8.1. Undecalcified samples: scanning electron microscopy

The images of the whole defect were taken using the backscattered electrons using a Stereoscan S360 (Leica/Cambridge Instruments). Image analysis was performed for the different images with an interactive software (Leica LAS v4.0, Leica). The region of interest (ROI) was defined as the outer limit where the material was in contact with the trabecular bone. The material (microspheres or calcium phosphate cements), empty spaces and different types of bone were distinguished by their morphology and the different contrast levels. The areas of new bone formation were measured, and normalized by the area of the defect.

#### 2.8.2. Decalcified samples

Stained histological sections were scanned using a light microscope (Leica DM2500, Leica). Five different regions were scanned and cell count was performed in four different fields.

### 2.9. Statistical Analysis

Statistical analysis was carried out with significance of 5% or less. One-factor analysis of variance (ANOVA) with Fisher’s post-hoc test was conducted. The data are expressed as mean value ± standard error.

## Results

### 3.1. Material characterization

**Fig 1:**
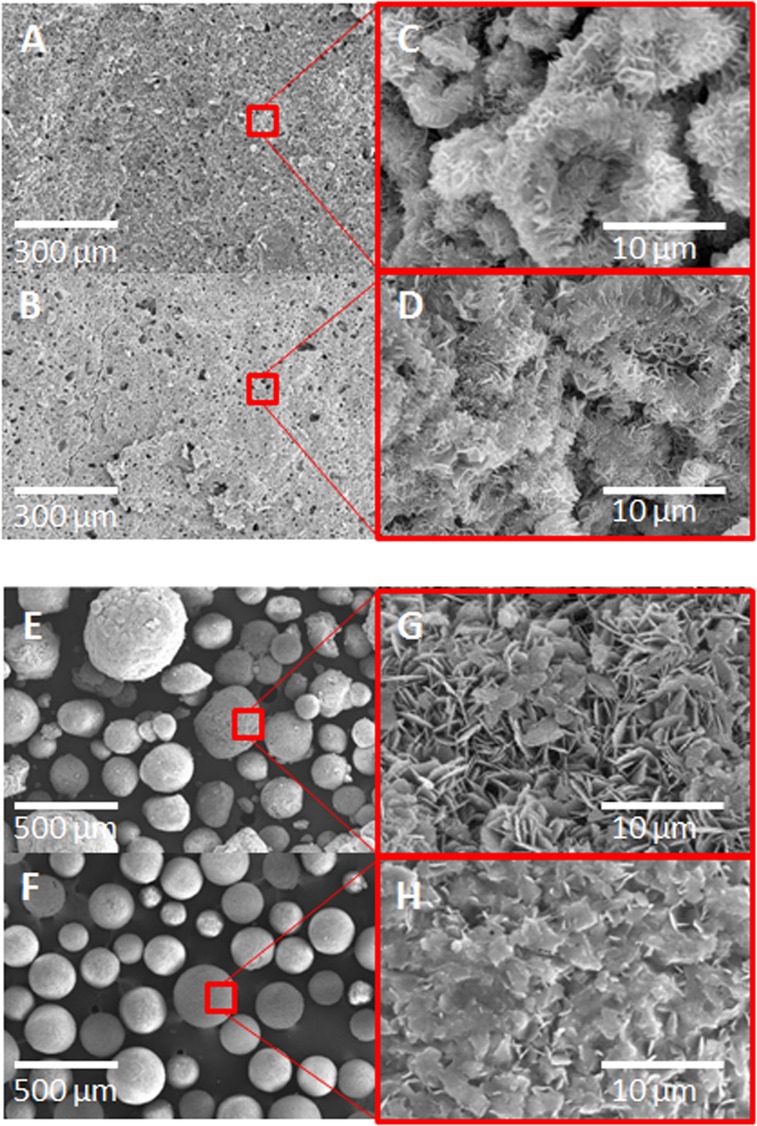
Scanning electron micrographs of the 4 implanted materials. CPC (A-D) and MS (E-H), in the absence (A,C,E,G) or presence (B,D,F,H) of collagen. (C,D,G,H) Microstructural morphology of the calcium deficient hydroxyapatite produced after hydrolysis of the starting α-TCP powder. The pristine CPC (C) and the coll-CPC (D) presented identical morphology, having typical hydroxyapatite crystals. The MS presented similar crystal morphology with bigger crystal domains (G). In the case of coll-MS collagen was exposed in the surface of the microspheres (H).

Fig 1 shows the SEM morphology for the different materials studied. Similar microstructures were observed for CPC (Fig 1A and 1C) and coll-CPC (Fig 1B and 1D), with the typical entangled hydroxyapatite crystals (Fig 1C and 1D). In contrast, the addition of collagen introduced significant differences in the morphology of the microspheres obtained from the corresponding ceramic slurries. Whereas the size range of the different microspheres was similar, since in both cases a sieving between 100 and 400 μm was applied, the coll-MS (Fig 1F) exhibited higher sphericity as well as a more homogenous size distribution than the MS (Fig 1E). At higher magnifications, the hydroxyapatite platelets were clearly visible on the surface of the MS (Fig 1G), similar to those observed in the CPC, although bigger in size. In contrast, in the surface of the coll-MS the presence of collagen was visible between the inorganic crystals (Fig 1H).

**Fig 2:**
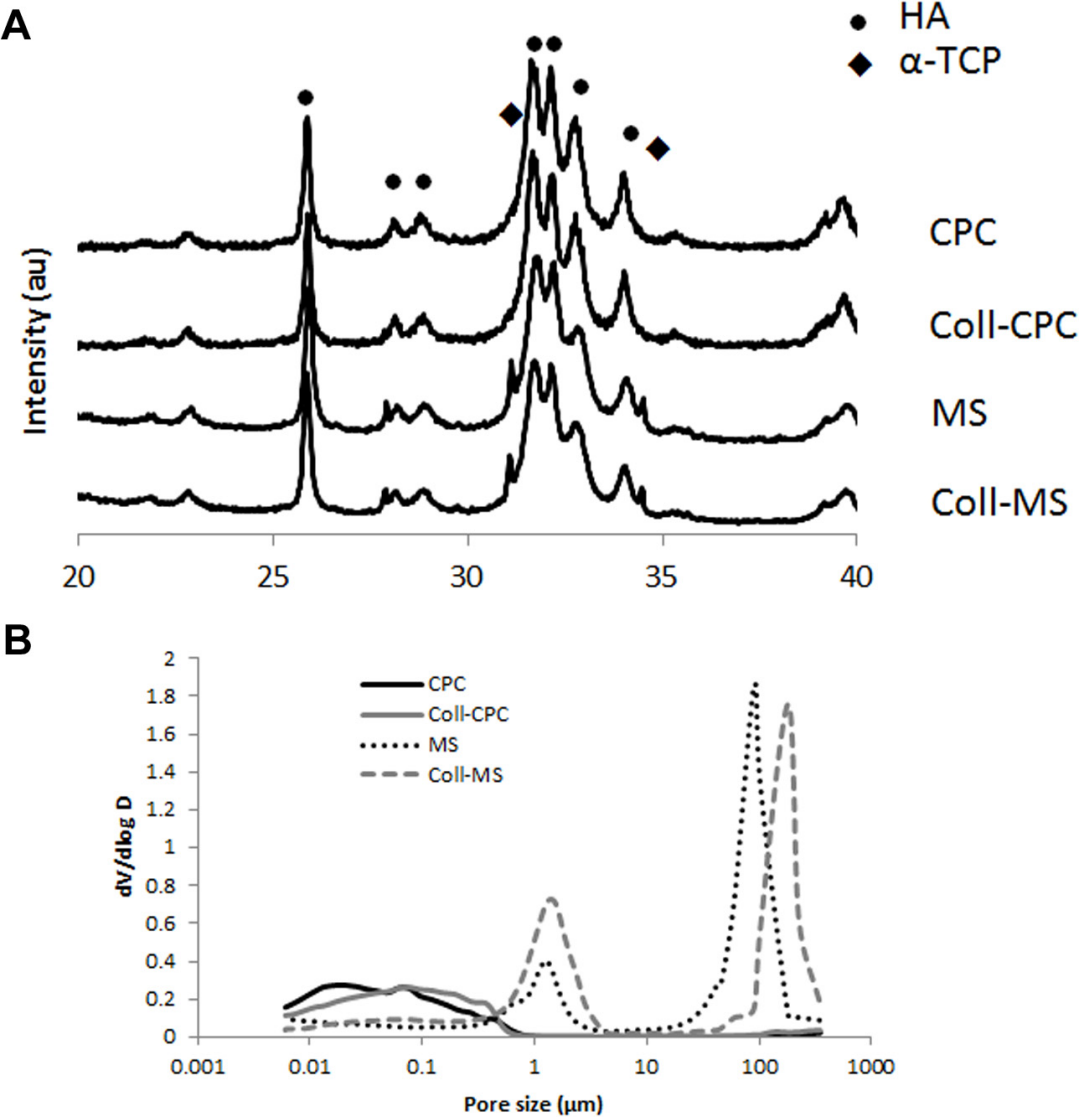
(A) X-Ray diffraction pattern of the different samples showing the complete transformation of the initial reagents into hydroxyapatite after 7 days immersion in water. The MS presented small remnants of unreacted α-TCP. (B) Pore size distribution of the studied samples obtained by mercury intrusion porosimetry.

As shown by the XRD diffractions patterns (Fig 2A), the presence of collagen did not hinder the transformation of α-TPC into a calcium deficient hydroxyapatite, although the MS presented small remnants of unreacted α-TPC in the structure. The Ca/P molar ratio of the resulting hydroxyapatite was 1.5, as the original α-TPC [9]. Furthermore, the diffraction peaks in the CPC and coll-CPC samples were slightly narrower than those of the MS and coll-MS samples. The pore size distribution in the different materials is displayed in Fig 2B. CPC and coll-CPC presented nanometric pores with a broad pore size distribution, ranging between 0.006 and 0.6 μm. In contrast, the microspheres presented a bimodal pore size distribution, with a narrow peak centered at 1 μm, corresponding to the pores in the microspheres, and a peak centered at 100 μm and 180 μm for the coll-MS and the MS respectively, which was related to the spaces between microspheres.

### 3.2. Clinical handling and radiographical assessment

**Fig 3:**
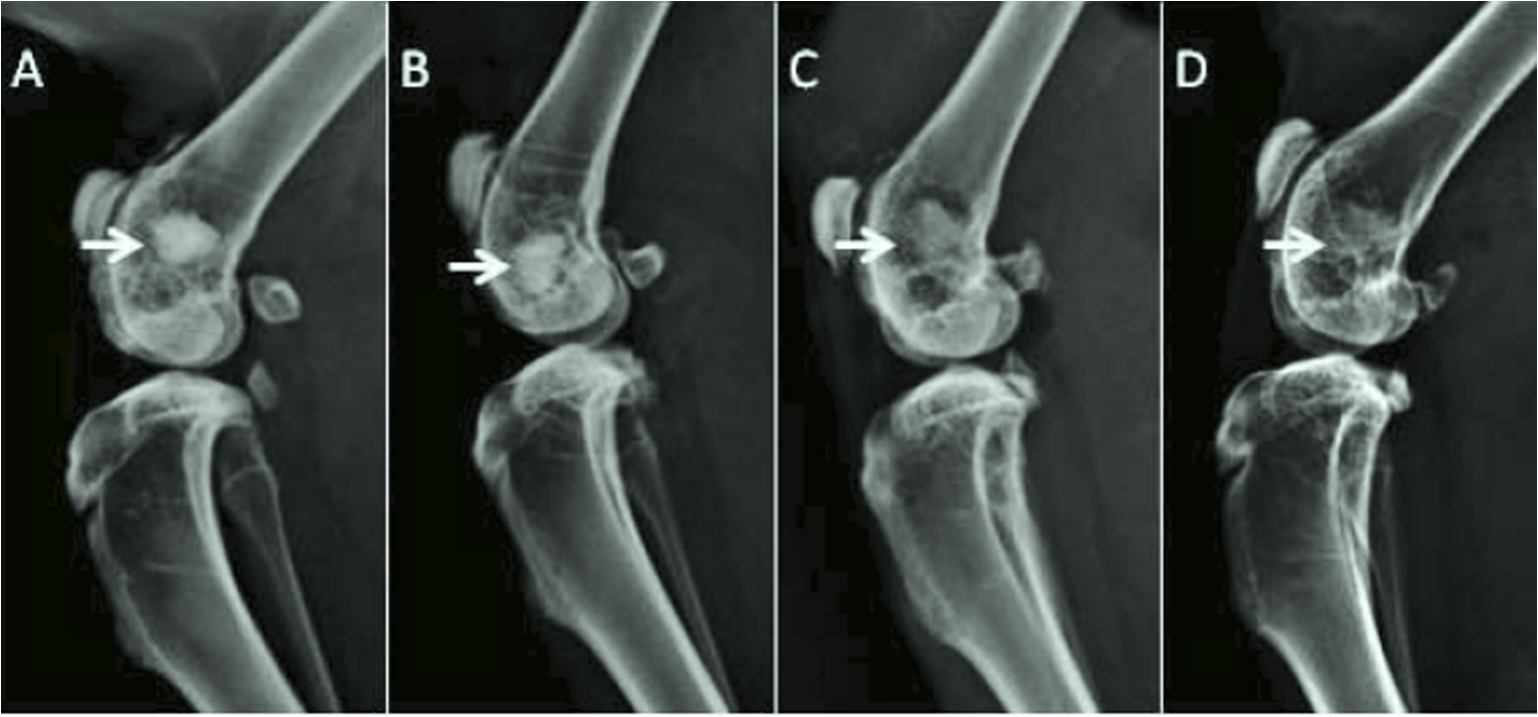
Radiographical assessment of the different samples implanted in the rat femur after 3 month implantation. (A) CPC, (B) coll-CPC, (C) MS and (D) coll-MS. The CPC samples (A,B) clearly showed evidence of material remaining in the site of implantation, whereas the microspheres (C,D) were less visible at the site of implantation.

No tendency to particle dispersion was observed intraoperatively by the surgeon during the handling of the microspheres-embebed clot when filling the defect. No clinical evidence of inflammatory response or adverse tissue reaction was found around implants and no toxic signs were observed for the experimental period. One month after implantation, the radiographical assessment showed complete consolidation of the samples in the defect site, with no detectable migration of the material, although the displacement of single microspheres could not be excluded. The radiological images after 3 months are shown in Fig 3. The materials started reducing their radiopacity, especially in the case of the MS (Fig 3C and 3D), with a loss of definition in the defect border as well as a reduction in the initial granular aspect.

### 3.3. Histology: decalcified samples

**Fig 4:**
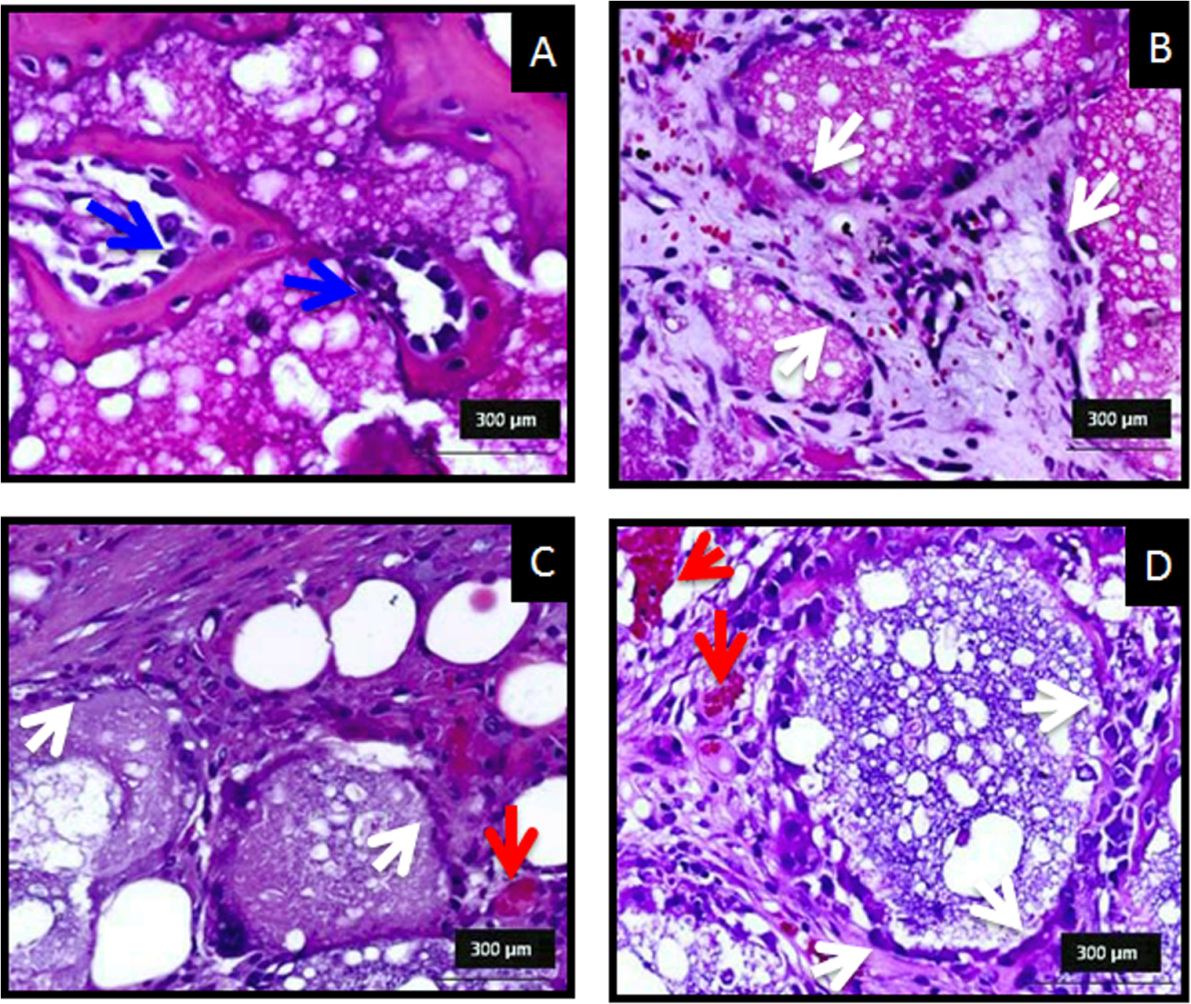
Histological analysis after 12 days post implantation, decalcified H&E stained sections of (A) CPC, (B) coll-CPC, (C) MS and (D) coll-MS. Sparse signs of inflammatory cells are visible, as red dots in the mesenchymal tissue invading the biomaterial, especially for the (B) coll-CPC, (C) MS and (D) coll-MS. The good biocompatibility is evidenced by the osteogenic cells aligning both at the interphase between the cement or the microspheres and the bone tissue (white arrows) and forming new bone lamellae. The lamellae in Fig 4A, suggest the formation of cutting cones (blue arrows). Blood vessels (red arrows) are visible around the microspheres (C and D).

After 12 days implantation, a small number of inflammatory cells were found in most of the histological sections, indicating the good biocompatibility of the different materials (Fig 4). Osteogenic cells were seen aligned at the interphase between the cement and bone tissue with new lamellar bone in the CPC and coll-CPC (Fig 4A and 4B). Similarly, osteogenic cells were observed surrounding each of the individual microspheres, and blood vessels were found in the rich mesenchymal tissue between the microspheres (Fig 4C and 4D). The higher surface available allowed for the penetration of a higher number of mesenchymal cells inside the defect, facilitating the alignment of osteogenic cells around the biomaterial as well as the formation of new blood vessels.

### 3.4. Backscattering scanning electron microscopy

**Fig 5:**
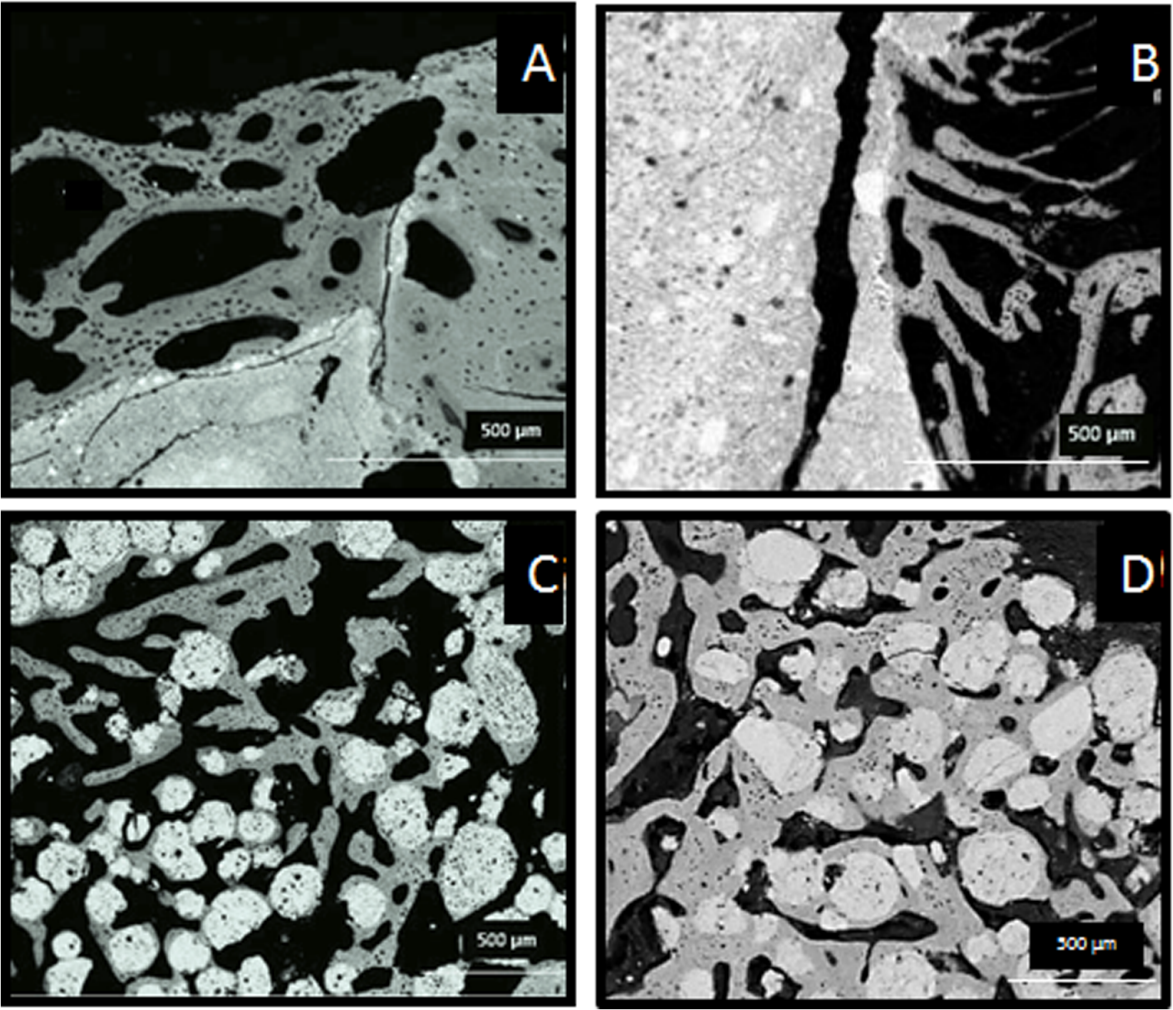
Backscattered electron micrographs of undecalcified section of the cements (A,B) and the microspheres (C,D) after 1 month implantation, in the absence (A,C) and presence (B,D) of collagen. Trabeculae constituted mostly by calcified chondroid tissue are visible in the periphery of the cements (A, B), whereas denser trabeculae with new bone covering the initial chondroid tissue were able to grow both on the surface of the microspheres and in the pores between them (C, D).

**Fig 6:**
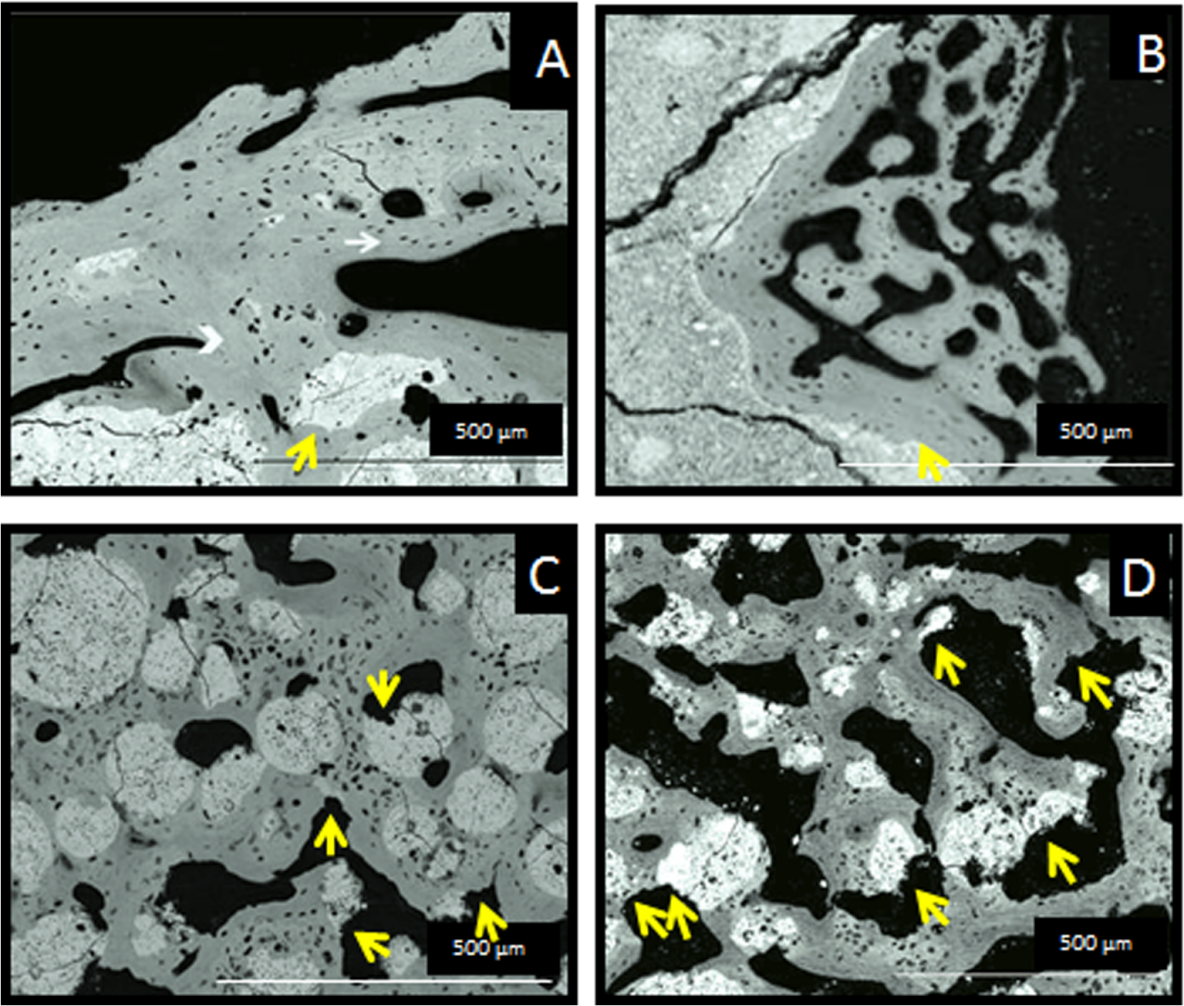
Backscattered electron micrographs of undecalcified sections of the cements (A, B) and the microspheres (C, D) after 3 months implantation, in the absence (A, C) and presence (B, D) of collagen. An increasing amount of lamellar new bone ingrowth (white arrow in A) upon a lesser amount of chondroid tissue (white arrowhead in A) were observed, showing the normal growth of the trabecular bone, following the same patterns mentioned in Fig 5. Noteworthy, the Howship’s lacunae (yellow arrows in A, B, C and D), indicative of the resorption of the material are more numerous around the collagen microspheres and the new bone formed around them (D).

SEM images after one and three months implantation are shown in Figs 5 and 6 respectively. New bone was observed growing on the periphery of the cements, in direct contact with its surface (Figs 5A and 5B and 6A and 6B), but the small-sized porosity hindered the penetration of new tissue within the material. A completely different result was observed for the microspheres (Figs 5C and 5D and 6C and 6D). The larger surface available and the open macroporosity generated between the individual microspheres enhanced cell penetration and bone ingrowth within the defect. The microspheres guided the formation of the new trabecular structure, becoming integrated in the new bone trabeculae. Calcified chondroid tissue was the principal component of the initial trabeculae surrounding all the biomaterials (Fig 5), but the coll-MS were covered by denser trabeculae, with a clear lamellar deposition (Fig 5D). At 3 months (Fig 6), the trabeculae around the biomaterial were consistently formed by lamellar bone, with only sparse remnants of the initial chondroid tissue (Fig 6B and 6C). Interestingly, some resorption was observed, more pronounced in the case of the MS and coll-MS (Fig 6C and 6D) with numerous Howship’s lacunae eroding both the biomaterial and the surrounding osseous tissues.

### 3.5. Histomorphometric analysis

**Fig 7:**
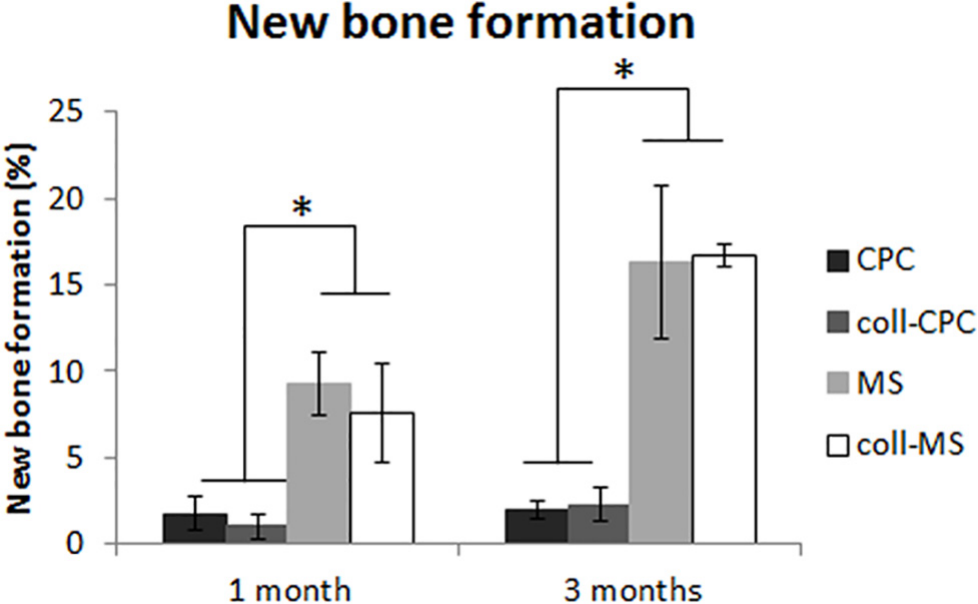
Histomorphometric quantification of the amount of new bone ingrowth. Significantly higher new bone ingrowth was found for the microspheres as compared to the cements. Furthermore, the amount of new bone formation was significantly increased from 1 to 3 months in the microspheres groups, and the same trend was found for the cements, although the increase was no statistically significant. * indicates significant differences between the groups connected with the line (p<0.05).

Fig 7 shows the amount of newly formed bone. After 1 month, the cements presented low values of new bone ingrowth (~1.7% and ~1.0% for CPC and coll-CPC respectively), compared to the microspheres (~9.3% and ~7.5% for MS and coll-MS respectively). After 3 months, the values were maintained low for the cements (~2% and ~2.3% for CPC and coll-CPC respectively), and were considerably increased for the microspheres (~16.3% and ~16.7% for the MS and coll-MS respectively), presenting nearly a two-fold increase respect to 1 month. The differences were shown to be significant between the cements and the microspheres, although there was no significant difference between the presence or absence of collagen for the different tested materials.

## Discussion

Apatitic calcium phosphate cements are widely used for bone regeneration. However, despite their excellent biocompatibility and osteoconductivity, their osteogenic potential is limited by the slow degradation, caused in part by the small size of their intrinsic porosity. Different strategies have been explored to overcome this limitation, such as the introduction of macroporosity in CPCs by different routes, like foaming the cement paste with the help of surfactant molecules or proteins [18–20], or adding soluble particles [21]. In this work we propose an alternative strategy, based on the fabrication of biomimetic calcium deficient hydroxyapatite microspheres, which have the same composition and microstructure of calcium phosphate cements because they are obtained by emulsification of calcium phosphate cement slurry. The strategy behind using the microspheres is the same as for the ceramics granules currently in the market, i.e., providing an interconnected pore network between the calcium phosphate particles, for the bone to invade. However, the composition and microstructure of these biomimetic microspheres are much closer to the biological apatite than that of sintered calcium phosphate granules, having also a much higher specific surface area. In fact, in a previous study it was shown that this type of low-temperature calcium phosphates, namely calcium deficient hydroxyapatite, can support bone ingrowth to a greater extent than ceramic calcium phosphates [22]. Moreover, the processing technique used in this study, both for the cements and the microspheres, was compatible with the combination with biopolymers, in our case collagen, which allowed obtaining collagen-calcium deficient hydroxyapatite composite materials [12]. The presence of collagen in the different materials did not hinder the hydrolysis of α-TCP to calcium deficient hydroxyapatite, and had a clear effect in facilitating the emulsifying process, due to the amphiphilic character of collagen and its subsequent surface active properties [7], as proved by the higher sphericity of the coll-MS (Fig 1E and 1F).

When comparing the two families of materials, namely cements and microspheres, it is important to keep in mind that the liquid to powder ratio used for each of them was different. The higher liquid to powder ratio in the case of the microspheres explains the larger pores observed by MIP (Fig 2B), shifting from ~0.006–0.6 μm for the cements to ~1 μm for the microspheres, which is consistent with previous studies [23]. On the other hand, the peak observed near 100 μm is related to the pore spaces between microspheres. This value represents the lower limit of the macropore size in the implanted blood-microspheres composite, since blood was not present in the MIP measurements. The bigger crystals observed in the microspheres compared to the cements, as shown in Fig 1 and indicated by the sharper diffraction peaks (Fig 2A), can also be related with the use of a higher liquid to powder ratio, which decreases the supersaturation of the liquid phase, promoting crystal growth instead of crystal nucleation.

The assessment of the implanted samples by histology after 12 days did not show any significant inflammatory response. Despite the use of bovine collagen, no immunogenic response was found. Interestingly, previous studies performed on spherical and sharp edged granules showed increase levels of inflammatory response in the sharp edged granules, therefore showing the advantage of using spherical particles as opposed to irregular granules [24].

Histological images indicated that both, cements and microspheres, were involved in the physiological processes of bone remodeling, as revealed by the presence of cutting cones in close contact with the material (Fig 4A) and the growth of the trabeculae formed around the materials (Figs 4 and 5). The presence of chondroid tissue, also described by Bailleul et al [25] as “chondroid bone”, in the initial trabeculae surrounding the biomaterial (Fig 5), as identified by back scattered SEM [26], cannot necessarily be interpreted as endochondral ossification. In fact, it has been demonstrated that chondroid tissue is an essential part of the human skull membranous ossification [27,28] and other repair processes [29,30], even if it can be present also in endochondral ossification. Moreover Bailleul et al. [25] described very thoroughly the evolution, morphological characteristics, and participation of chondroid tissue in endomembranous ossification in different species, as they have been described in the literature. All materials were shown to be extremely osteoconductive, as their surface was observed to be completely covered by new bone, as observed in previous studies with similar materials [22]. However, the intrinsic pore size of the cements was shown to be too small for cell penetration, and therefore bone was able to grow only in the periphery of the implanted material, being the amount of newly formed bone small, which is in agreement with previous studies [18,20]. A dramatic increase in bone formation (10-fold) was observed when the material was used in the form of microspheres instead of a continuous paste (Fig 7). The enhanced behavior of the microspheres in comparison to the cements can be attributed to the highly interconnected porous network created between particles, which allowed for the presence of the blood clot throughout the whole defect. It is well known that the cells and specific growth factors present in the fibrinous blood clot [31,32] play an essential role in bone regeneration. As previously mentioned, the gaps between the MS were found to be at least 100 μm, which has been shown to be an excellent interconnectivity value to allow bone tissue formation within calcium phosphate ceramics [33].

The values obtained for new bone deposited in the defect in presence of the microspheres are in the range of those reported in previous studies with calcium phosphate granules using the rabbit femoral condyle model [22, 34–37]. It is important to keep in mind, however, that it is not possible to establish direct comparisons, due to different experimental protocols (defect size, quantification method, etc.), or different characteristics of the materials used (granule size, use of carriers, etc.). It is well known, for instance, that granule size has an impact in bone formation [34,38], and that the results obtained when quantifying bone formation by microCT tend to be higher than those obtained by back scattering SEM [35,39]. Aguado *et al*. [36] reported around 14% new bone formation for beta-TCP granules after 1 month, measured by microCT. Moreover, they showed that the use of a hydrogel as a carrier, in this case hyaluronic acid, in addition to facilitating the handling of the granules, increased bone apposition, due to the fact that more granules were maintained in the surgical site. Castellani *et al*. [37] found that the amount of new bone when using biphasic calcium phosphate (BCP) granules in a fibrin gel was 12% after 2 months [37], whereas Le Nihouanen *et al*. [35] reported around 20% after 3 months. The amount of new bone reported by Bourgeois *et al*. [22] after only 3 weeks for BCP and calcium deficient hydroxyapatite granules combined with hydroxypropylmethylcellulose was larger, (around 18% for BCP granules and 24% for calcium deficient hydroxyapatite). This can be explained by the much smaller granule size (40–80 micrometers) [34,38].

No clear effect on bone formation was seen when collagen was incorporated in the materials. Although it was reported in previous *in vitro* studies that the incorporation of collagen in calcium phosphate cements and microspheres not only enhanced osteoblastic cell adhesion and proliferation [7,14,15], but also fostered the expression of proteins involved in osteogenesis and mineralization [7,15], no statistically significant differences in the amount of newly formed bone were found between the collagen-containing materials and their pristine counterparts. However, even though no quantification was performed, a higher erosion was observed in the coll-MS as compared to the MS (Fig 6D), which could be indicative of a faster degradation. The morphology and shape of the eroded surfaces found both in the bone and the MS resembled those of Howship’s Lacunae as was previously reported and was shown to be related with the osteoclastic activity of cells in the bone matrix [40]. This effect was not obvious in the cements, which can be related to the smaller porosity, together with the fact that the amount of collagen in the injectable cements was smaller than in the microspheres, due to the higher liquid to powder ratio used in the later (4.5 mg coll/g CPC for the injectable pastes and 8 mg coll/g CPC for the microspheres, which represents 0.45 and 0.8 wt% or collagen, respectively).

The smaller effect of collagen addition in the materials observed in this study can seem contradictory with other studies that have reported a significant enhancement of degradation when hydroxyapatite was combined with collagen [41–43]. However, it is important to keep in mind that in this study, both in the cements and the microspheres collagen was a minority phase. This was conditioned by the fact that collagen was incorporated in the liquid phase of the self-setting cement, and therefore the possibility to obtain a collagen solution with adequate viscosity limited the total amount that could be added. When collagen/hydroxyapatite composites are obtained by other routes, much higher amounts of collagen can be incorporated in the material, and therefore the effects are much more visible.

## Conclusions

The use of biomimetic microspheres obtained by emulsification of a calcium phosphate cement proved to be a good strategy that significantly increased the amount of bone ingrowth, compared to completely filling the defect with a calcium phosphate cement paste. The incorporation of collagen into the calcium phosphate cement did not have a significant effect on new bone formation.
